# 

**Published:** 1810-09

**Authors:** 


### CORRESPONDENCE.

Communications are received from Dr. J. Bradley and Mr. White, which will appear in our next Number.

